# Remote Patient Monitoring of Blood Pressure Is Feasible Poststroke and Can Facilitate Triage of Care

**DOI:** 10.1089/tmr.2022.0004

**Published:** 2022-08-02

**Authors:** Jenna M. Tosto-Mancuso, David Putrino, Jamie Wood, Laura Tabacof, Erica Breyman, Leila Nasr, Nicki Mohammadi, Neha S. Dangayach, Christopher P. Kellner

**Affiliations:** ^1^ Department of Rehabilitation and Human Performance and Icahn School of Medicine at Mount Sinai, New York, New York, USA.; ^2^ Department of Neurosurgery, Icahn School of Medicine at Mount Sinai, New York, New York, USA.

**Keywords:** remote monitoring, stroke, telehealth, mHealth, blood pressure

## Abstract

**Background and Purpose:**

Strict blood pressure (BP) control is a universally accepted therapeutic intervention in the prevention of secondary stroke, yet this remains difficult when patients return home postinjury. This study aimed to investigate the application of the remote patient monitoring (RPM) of BP in patients after stroke, or who were at immediate risk of stroke, and the subsequent outcomes relating to triage and escalation of care.

**Methods:**

This was a single-center proof-of-concept study. Participants were patients aged 18 years and older with a diagnosis of stroke or who were at immediate risk of stroke. Patients were enrolled into the precision recovery program (PRP) and asked to assess their BP and heart rate daily and enter values into a MyCap application for the RPM program. These data were reviewed daily by an assigned PRP clinician, and weekly Zoom meetings were held with the patient. Care was triaged and escalated to a physician as indicated.

**Results:**

Twelve patients (5 [42%] female, aged mean [range] 63 [43–84] years) met the inclusion criteria and continued in the program for median (range) 136 (8–227) days. The median (range) number of excursions of BP above limits per participant was 19 (0–79) for systolic and 36 (0–104) for diastolic. A total of 16 triage events (median [range] 1 [0–3]) were initiated for escalation of care.

**Conclusions:**

This study demonstrated that RPM is feasible in patients poststroke or at immediate risk of stroke, and facilitates the triage of care when BP is elevated above recommended limits.

## Introduction

Stroke is the third leading cause of long-term disability and the second leading cause of mortality worldwide.^1^ In the United States, a stroke occurs every 40 sec, resulting in ∼795,000 instances of stroke each year.^2^ Of these, almost one quarter are considered recurrent strokes.^2^ After discharge from the health system after acute stroke care, there is limited structured monitoring of vital signs and symptom evolution, creating a disconnect between patient and health system that may pose safety risks. Strict blood pressure (BP) control is a universally accepted therapeutic intervention in the prevention of secondary stroke, yet this remains difficult when patients return to the home setting.^3–7^

Traditional models of stroke care lack an integration of remote patient monitoring (RPM) to assist with the management of BP in the home setting, including assessment of patient compliance with postdischarge recommendations. Although in-person follow-up visits are generally scheduled on a tri-annual basis, these encounters fail to capture day-to-day BP variability. More regular measures are crucial to the success of BP management strategies in individuals after stroke.^8–10^

A lack of structured monitoring of physiological data (i.e., BP, heart rate [HR]) or symptom evolution (i.e., motor control, speech and language, functional status, cognitive status) makes it difficult to identify precipitating factors of clinical deterioration at an earlier stage. This lack of early detection may potentially result in development of more severe sequelae including worsening neurological presentation.^11–13^

RPM is the regular transmission of health data from the patient's location to a health care provider (usually at a hospital or clinic).^14^ Telehealth, in the form of RPM, presents a potential solution for improved monitoring of BP poststroke. Although the use of RPM has been used more widely in older adults with cardiac, respiratory, and metabolic conditions, the use of this technology remains limited in poststroke cohorts.^15–18^

Previous study investigated the use of digital BP measurement and role of behavioral intensification strategies for improving management of BP in the home.^19,20^ These studies primarily focused on the feasibility of behavioral intensification to promote adherence to remote BP monitoring without investigating clinical outcomes. Therefore, this study aimed to investigate the application of the RPM of BP in patients after stroke, or those with elevated risk of stroke, and the subsequent outcomes relating to triage and escalation of care.

## Materials and Methods

### Study design

This was a single-center proof-of-concept study conducted between January 2020 and August 2020. Approval was obtained by the institutional review board (IRB no. 20-01653) for retrospective review of all clinical data for the precision recovery RPM program. Informed consent was waived by the institutional review board as precision recovery program (PRP) was a part of clinical care.

### Participants

Participants were patients enrolled in the PRP at Mount Sinai Health System during the 7-month study period. Referrals to the PRP were received from the health system's inpatient and outpatient neurosurgical and neurology departments.

Requirements for participation in the program were being aged >18 years with diagnoses of stroke or immediate risk of stroke, and having access to a smart device or dial up telephone. Patients who met inclusion criteria were referred to the program by message to the PRP clinicians through the health system's electronic medical record (EPIC), or through e-mail referral from the referring physician. Patients were contacted by the PRP team, and scheduled for an onboarding session through Zoom (Zoom Video Communications, Inc., San Jose, CA).

### Precision recovery onboarding

During onboarding, a PRP clinician (physical therapist, nurses, physician assistants) collected baseline information including patient history including date of stroke, recommended BP parameters as indicated by the referring physician, and medications prescribed, and guided the patient through downloading the MyCap smartphone application. After this, the patient was asked to take his or her BP using an Omron digital BP cuff provided to the patient (Omron Healthcare Inc., Lake Forest, IL).

Devices were mailed to the patient before the initial onboarding session or provided to the patient at time of discharge if being referred to the program from the in-patient setting. During this session, a PRP clinician supervised the patient entering BP and HR data into the daily symptom log within the MyCap application. Those without access to a smart device were advised that they would be contacted daily by a team member of the PRP to verbally obtain the requested data.

### Precision recovery MyCap application

Members of the PRP developed a data collection tool using Research Electronic Data Capture (REDCap) electronic data capture tools hosted at Mount Sinai Health System.^21,22^ REDCap is a secure web-based software platform designed to support data capture for research studies. MyCap is an external module of REDCap that facilitates the deployment of REDCap data collection using a smart device, through a proprietary application programming interface.^21,22^

### Home monitoring and triage

Patients were asked to assess their BP and HR daily and enter values into the MyCap application. These data were reviewed daily by an assigned PRP clinician. Patients were encouraged to enter BP at an established time daily for consistency. A weekly meeting through Zoom was also scheduled at a time convenient to the patient. During this meeting, the BP data were discussed with the patient including interpretation of BP trends and review of BP-relevant medications. The weekly Zoom meetings were ∼20 min in length. Relevant outcome measures and a neurological screen were also assessed during this time.

Parameters for acceptable systolic and diastolic BP were set by the referring neurosurgeon or the patient's primary care physician. This was predominantly set at measures of no greater than 130 mmHg systolic and 80 mmHg diastolic, with occasional exceptions were allowed according to acceptable clinical management. A triage episode was initiated if (1) BP exceeded the defined limits and the PRP clinician determined this was outside of observed clinical trends and/or (2) a new onset neurological sign was observed by the clinician during the weekly meeting. Triage consisted of the PRP clinician contacting the patient and arranging an immediate review through Zoom.

If requested by the PRP clinician, a PRP physician (neurologist or neurosurgeon) joined the clinician to complete a medical assessment. After the triage episode, the physician made recommendations regarding whether to continue daily RPM or to escalate care. Escalation of care included referral to the emergency room, or referral back to the patient's referring physician. Throughout the PRP, it was reiterated to patients that RPM was not an acute care service, that is, patients were advised to present to the nearest emergency department or contact 911 in the event of a medical emergency.

### Clinical data collected

#### Demographics and neurological event history

Measurements were collected at enrollment and included broad medical and surgical history, and details of the neurological event leading to referral to the PRP. Retrospective chart review was completed to obtain data including age, date of acute injury, social history, patient gender, race, stroke type (ischemic or hemorrhagic), and the intervention required at time of acute injury.

#### BP, HR, and triage

Daily BP data were obtained through the MyCap application. Trends and excursions above defined BP limits were monitored and collated at the end of the program. The number of triage events and triage outcomes was collated at the end of the program. Adherence to the daily reporting of data were calculated at the end of the program.

#### Neurological screen

A neurological screening assessment was completed at onboarding and at subsequent weekly meetings with the PRP clinician. The physical and cognitive assessment included evaluation of gross motor control (by way of bilateral shoulder flexion, bilateral hip flexion), coordination and dysmetria through finger to nose assessment, facial symmetry assessment through assessment of smile, and cognition by assessment of three orientation questions: (1) what is your name, (2) how old are you, and (3) who is in the room with you? This assessment was recorded through video for retrospective kinematic analysis. The feasibility of regularly completing the neurological screen through telehealth was assessed by calculating the completion rate at the end of the program.

### Analyses

Statistical analyses were undertaken with Stata (StataCorp, Stata Statistical Software Release: V.14). Data were retrieved from the REDCap database before analyses being conducted. Data were analyzed using descriptive statistics.

## Results

Twelve patients (5 [42%] female, aged mean [range] 63 [43–84] years) who met the inclusion criteria were referred to the PRP (Table 1) and continued in the program for median (range) 136 (8–227) days. Reasons for discontinuing the PRP early were a requirement for surgery (*n* = 1, ceased PRP after 8 days) and for moving outside of the United States (*n* = 1, ceased PRP after 47 days). Adherence to the daily reporting of measurements was mean (range) 70 (41–100)%. The common reasons for failing to report measurements on any day included patient forgetting to take BP and patient forgetting to enter data. No reports of difficulties using the mobile application were noted.

**Table 1.** Participant baseline demographic data (*n* = 12)

Demographic data	
Female	5 (42)
Age, years, mean (range)	63 (43–84)
Stroke type^a^	
Ischemic	4 (33)
Hemorrhagic	7 (58)
Acute management	
Medical management only	6 (50)
SCUBA	2 (17)
Other^b^	4 (33)
Comorbidities	
Hypertension	10 (83)
Cardiac disease	5 (42)
Hypercholesterolemia	4 (33)
Type 2 diabetes mellitus	2 (17)
Smoking history	5 (42)
Race	
LatinX	4 (33)
White	4 (33)
Black or African American	3 (25)
Asian	1 (8)
English as primary language	12 (100)

Data are presented as *n* (%) unless otherwise stated.

^a^ One participant was being monitored due to essential hypertension and risk of stroke.

^b^ Other management includes antithrombotic/antihypertensive therapy, coil embolization, stenting.

SCUBA, stereotactic intracerebral hemorrhage underwater blood aspiration.

### BP, HR, and triage

**FIG. 1.**
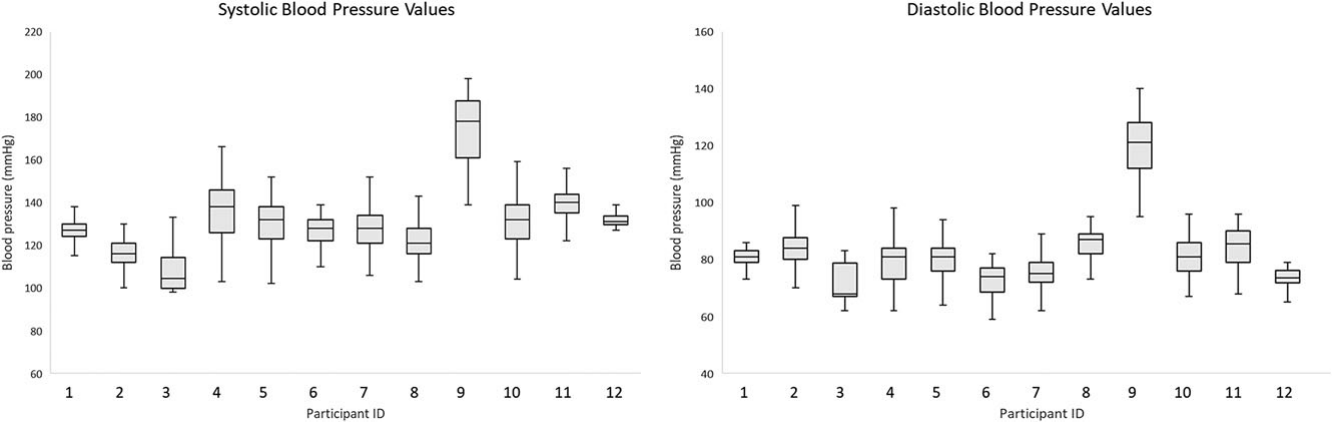
Participants' mean systolic and diastolic BP data (*n* = 12). BP, blood pressure.

**FIG. 2.**
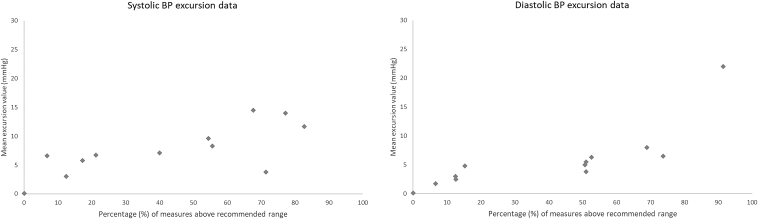
Participants' systolic and diastolic BP excursion data (*n* = 12).

The majority of patients (*n* = 9, 75%) had BP safety limits set at 130/80 mmHg. The other three patients' BP limits were set at 140/80, 130/90, and 160/100 mmHg. Participant BP data are shown in Figure 1. The median (range) number of excursions above BP limits per participant was 19 (0–79) for systolic and 36 (0–104) for diastolic (Fig. 2). The mean (range) HR was 70 (59–82). A total of 16 triage events (median [range] 1 [0–3]) were initiated for escalation of care. Of these, 11 were triaged to the referring physician, 2 were triaged to urgent care/emergency department, and 1 was triaged to the patient's primary care physician.

### Neurological screen

The neurological screen was successfully completed during all 208 (100%) of the weekly Zoom meetings. During one assessment, a deterioration in neurological signs (facial symmetry) was detected and resulted in an urgent triage of care to the neurologist and subsequent repeat of neuroimaging. No other episodes of deterioration were observed during these screens.

## Discussion

In this proof-of-concept study, RPM was feasible and implemented in a small cohort of patients to assist in the observation of BP. All but one of the patients were poststroke, with one undergoing monitoring for unstable essential hypertension. There was, on average, good adherence to the daily reporting of BP, and in certain cases this led to the triage of care to the referring physician or the emergency department. There were, however, only two presentations to the emergency department, and, therefore, RPM may have assisted in decreasing the burden on acute care services through the increased contact with the PRP team. The PRP clinicians were also able to successfully conduct neurological screens remotely, which resulted in the evolution of neurological symptoms being detected in one patient.

There were several limitations noted within this study. As BP was not continuously monitored, fluctuation in BP over the course of a 24-h period was possible. To preserve reporting adherence, daily symptom reporting was deemed sufficient without overburdening the patients. The adherence to daily reporting (70%) was considered good, however, this was variable and reduced in some of the patients enrolled in the program over a longer timeframe. Despite this, sufficient measures were collected to allow adequate BP monitoring over time (i.e., at least every other day), reinforced with the relatively small number of triage required.

The number of total patients was small, yet it is anticipated that the use of RPM in this population will continue to grow beyond this study given the initial success of the program.

As a pilot program of the initial version of the precision recovery application, there are items that can be improved upon. In the updated version of the application, visualized elements will be made available to both clinicians and patients to better view and understand trends in BP. Furthermore, integration into the health system's electronic medical record will allow for optimized continuity of care.

The remote monitoring of BP allowed PRP clinicians to review the number and size of excursions above the recommended limits set by the referring physicians. Excursion data were variable between patients, though values were predominantly <15 mmHg on average. One participant, who had an ischemic stroke on a background of essential hypertension, reported systolic and diastolic BP measurements above the recommended limits the majority of the time despite these being set at 160 and 100, respectively. Despite this, care of this participant only required triage to the referring physician on one occasion.

The number of triage events among all patients was low in comparison with the number of total excursions of BP above recommended limits. This highlights the important role of the PRP clinicians in synthesizing the reported data and using this to assist clinical decision making. The role of telehealth in BP management is supported by the American College of Cardiology and American Heart Association, with RPM and input from health care providers demonstrated to be more effective at treatment modification than patient self-monitoring alone.^11^ Similarly, recent work investigating the feasibility of RPM of BP in patients after ischemic stroke further emphasized the need for human supervision of the captured data to improve the continuity of care.^5^

The use of a neurological screen is considered an important component of poststroke assessment. It is used to identify changes in clinical status and evolution of neurological symptoms. In this RPM program, neurological screening was successfully completed in Zoom meetings with the PRP clinician. Remote neurological assessment of physical function and cognition has previously been demonstrated to be feasible and efficacious.^16^ The use of a neurological screen in this study was justified with the identification of worsening facial droop in one patient, with care being escalated urgently for further assessment.

The level of adherence observed in this study was comparable with past work investigating RPM interventions that involved daily reporting.^11^ In general, levels of adherence reduced in the patients who were required to measure their BP for several months, indicating a level of reporting fatigue. Given the likelihood for this to continue to occur, consideration should be given for a reduction in the frequency of reporting (i.e., three times per week) in patients who remain in the PRP for extended periods of time, and are no longer considered to be in the acute phase of their poststroke care.

## Summary

This proof-of-concept study demonstrated that RPM is feasible poststroke, or in those at immediate risk of stroke, and facilitates the triage of care when BP is elevated above recommended limits. The regular review of BP data, and patients, by PRP clinicians, provided an additional layer of clinical interpretation that was integral in ensuring triage was utilized appropriately. This potentially reduced the burden on acute care services by preventing unnecessary emergency department visits that may have occurred if patients were self-monitoring. RPM of BP in conjunction with regular clinical telehealth reviews should be considered by neurological teams to assist in the effective triage of care.

## Abbreviations Used

BP: blood pressure
RPM: remote patient monitoring
PRP: precision recovery program
HR: heart rate
REDCap: research electronic data capture
